# Association of Primary Care Visit Length With Potentially Inappropriate Prescribing

**DOI:** 10.1001/jamahealthforum.2023.0052

**Published:** 2023-03-03

**Authors:** Hannah T. Neprash, John F. Mulcahy, Dori A. Cross, Joseph E. Gaugler, Ezra Golberstein, Ishani Ganguli

**Affiliations:** Division of Health Policy and Management, School of Public Health, University of Minnesota, Minneapolis; Division of Health Policy and Management, School of Public Health, University of Minnesota, Minneapolis; Division of Health Policy and Management, School of Public Health, University of Minnesota, Minneapolis; Division of Health Policy and Management, School of Public Health, University of Minnesota, Minneapolis; Division of Health Policy and Management, School of Public Health, University of Minnesota, Minneapolis; Harvard Medical School, Boston, Massachusetts; Brigham and Women’s Hospital, Boston, Massachusetts

## Abstract

**IMPORTANCE:**

Time is a valuable resource in primary care, and physicians and patients consistently raise concerns about inadequate time during visits. However, there is little evidence on whether shorter visits translate into lower-quality care.

**OBJECTIVE:**

To investigate variations in primary care visit length and quantify the association between visit length and potentially inappropriate prescribing decisions by primary care physicians.

**DESIGN, SETTING, AND PARTICIPANTS:**

This cross-sectional study used data from electronic health record systems in primary care offices across the US to analyze adult primary care visits occurring in calendar year 2017. Analysis was conducted from March 2022 through January 2023.

**MAIN OUTCOMES AND MEASURES:**

Regression analyses quantified the association between patient visit characteristics and visit length (measured using time stamp data) and the association between visit length and potentially inappropriate prescribing decisions, including inappropriate antibiotic prescriptions for upper respiratory tract infections, coprescribing of opioids and benzodiazepines for painful conditions, and prescriptions that were potentially inappropriate for older adults (based on the Beers criteria). All rates were estimated using physician fixed effects and were adjusted for patient and visit characteristics.

**RESULTS:**

This study included 8 119 161 primary care visits by 4 360 445 patients (56.6% women) with 8091 primary care physicians; 7.7% of patients were Hispanic, 10.4% were non-Hispanic Black, 68.2% were non-Hispanic White, 5.5% were other race and ethnicity, and 8.3% had missing race and ethnicity. Longer visits were more complex (ie, more diagnoses recorded and/or more chronic conditions coded). After controlling for scheduled visit duration and measures of visit complexity, younger, publicly insured, Hispanic, and non-Hispanic Black patients had shorter visits. For each additional minute of visit length, the likelihood that a visit resulted in an inappropriate antibiotic prescription changed by −0.11 percentage points (95% CI, −0.14 to −0.09 percentage points) and the likelihood of opioid and benzodiazepine coprescribing changed by −0.01 percentage points (95% CI, −0.01 to −0.009 percentage points). Visit length had a positive association with potentially inappropriate prescribing among older adults (0.004 percentage points; 95% CI, 0.003–0.006 percentage points).

**CONCLUSIONS AND RELEVANCE:**

In this cross-sectional study, shorter visit length was associated with a higher likelihood of inappropriate antibiotic prescribing for patients with upper respiratory tract infections and coprescribing of opioids and benzodiazepines for patients with painful conditions. These findings suggest opportunities for additional research and operational improvements to visit scheduling and quality of prescribing decisions in primary care.

## Introduction

Time is a scarce and valuable resource in primary care, with the average visit lasting 18 minutes.^1^ By a recent estimate, primary care clinicians would require 27 hours per day to provide all guideline-recommended preventive, chronic disease, and acute care to a typical patient panel.^2^ While there is global variation in primary care visit length,^3^ recent growth in visit content (eg, diagnoses recorded and medications prescribed) has outpaced growth in visit length,^4,5^ suggesting that time available per health concern may be decreasing over time.^6^ In surveys, patients routinely report needing more time with their primary care physician,^7,8^ and visit length is one of the most prominent factors associated with patients’ satisfaction with their care.^9,10^ Physicians also want more time with their patients and frequently report feeling rushed during visits.^11–13^

It is widely believed that shorter visits are associated with lower-quality care for patients.^14,15^ In particular, there is concern that clinicians make less-appropriate prescribing decisions in shorter visits since it takes time to make diagnoses, discuss existing treatment regimens, identify potential medication conflicts, and deprescribe as necessary.^16^ Clinicians may view some prescriptions (eg, opioids, antibiotics) as quick fixes when discussion of alternatives (eg, physical therapy, watchful waiting) would take additional time and effort or as a strategy to resolve a tense patient interaction.^17–19^

Yet, evidence on the association between visit duration and quality of care is limited and mixed. One study using national survey data found that providing recommended counseling or screening required additional time, but appropriate medication prescribing for chronic conditions was not associated with visit duration.^20^ Another study using the same national survey data found that upper respiratory tract infection visits that included an antibiotic prescription were shorter than visits without an antibiotic prescription.^21^ Other research using direct observation techniques found more complete discussion of new prescription medications during longer visits.^22^ Finally, some studies have documented an association between time pressure and the provision of likely low-value prescribing but not for all outcomes studied.^23,24^ To our knowledge, none of these studies accounted for known practice differences between clinicians in their baseline propensity for visit length and prescribing decisions.

Using a multistate sample of electronic health record (EHR) data, we first examined patient clinical and sociodemographic characteristics associated with visit length. Controlling for these characteristics, we then examined within-physician changes in potentially inappropriate prescribing decisions, including inappropriate antibiotic prescribing, coprescribing opioids and benzodiazepines, and potentially inappropriate prescribing for older adults as a function of primary care visit duration. Results from these analyses may inform policy makers and health system leaders as they balance visit volume pressures and the need to deliver high-quality care.

## Methods

Because only deidentified administrative data were used, this cross-sectional study was deemed not to be research involving human participants and therefore was exempt from informed consent requirements and institutional review board review by the institutional review board at the University of Minnesota. This study followed the Strengthening the Reporting of Observational Studies in Epidemiology (STROBE) reporting guideline.

### Study Population

We used a subset of claims and EHR data from athenahealth Inc, a cloud-based health care information technology company that provides physician practices with medical billing, practice management, and EHR services. These data have been used in prior work related to visit length measurement and prescribing behavior.^1,24,25^

The study sample included visits for adult patients seeing primary care physicians (defined as those with internal medicine, family practice, and general practice specialties) across the US who used the full suite of athenahealth services (ie, billing management and EHR) in calendar year 2017. Using previously validated methods,^1,25^ we excluded visits without reliable measures of observed duration (eFigure 1 in Supplement 1 gives additional detail on sample construction). We used visit subsamples to assess specific prescribing outcomes: visits with a diagnosis of upper respiratory tract infection (for the inappropriate antibiotic prescribing outcome), visits with a pain-related diagnosis (for the coprescribing of opioids and benzodiazepines outcome), and visits for adults aged 65 years or older (for the potentially inappropriate prescribing among older adults outcome) (the Table gives patient and visit characteristics of each sample, and eTable 1 in Supplement 1 gives a list of *International Statistical Classification of Diseases and Related Health Problems, Tenth Revision* [*ICD-10*] diagnosis codes and medications used to define subsamples).

### Visit Length

We measured visit length using time stamps, which document clinicians’ actions in the EHR across stages of a patient encounter from check-in and intake through face-to-face encounter, checkout, and signoff. Typically, once staff members have completed check-in (eg, confirming insurance coverage), a medical assistant conducts the intake assessment (eg, vital signs and medication reconciliation). Following intake, the physician clicks “Go to Exam” to start the visit (eg, taking patient history, performing a physical examination, and placing orders). At the end of the visit, the physician closes the examination stage to advance the encounter to the checkout stage. To measure visit length, we used previously published methods of processing time stamps recorded during the face-to-face examination stage of each primary care visit, which encompasses the interaction between patient and physician.^1,25^

### Potentially Inappropriate Prescribing Outcomes

We examined 3 outcomes representing inappropriate or potentially inappropriate prescribing decisions: inappropriate antibiotics for upper respiratory tract infections, coprescribing of opioids and benzodiazepines, and potentially inappropriate prescribing for older adults. For inappropriate antibiotic prescribing, we implemented a widely used definition relying on the presence of an antibiotic prescription linked by exact patient identifier, physician identifier, and date to a visit with a primary diagnosis of upper respiratory tract infection.^26^ Similarly, we defined opioid and benzodiazepine coprescribing as a visit with a pain-related primary diagnosis and both an opioid and a benzodiazepine prescription linked to the visit.^24^ As a sensitivity analysis, we repeated this prescribing outcome among visits with both a pain-related primary diagnosis and an anxiety diagnosis. Finally, we identified all visits for adults aged 65 or older that were linked to prescriptions for medications listed by the 2019 updated Beers criteria^27^ (ie, a consensus statement from the American Geriatrics Society on potentially inappropriate medications for older adults) as having a strong recommendation of avoid based on high-quality evidence. If a prescription was linked to the visit, this meant that the prescription was newly ordered, refilled, or confirmed at the visit. As with past work,^24^ we linked prescriptions to visits by exact patient identifier, physician identifier, and date.

### Additional Covariates

We used submitted insurance claims and structured EHR data based on patient self-report to collect visit-level data on patients’ age, sex, marital status, race and ethnicity (collected via patient self-report by medical practices; Hispanic, non-Hispanic Black, non-Hispanic White, other [American Indian or Alaska Native, Asian, and Native Hawaiian or Other Pacific Islander], and missing), primary insurer (ie, commercial, dual eligible [for Medicare and Medicaid], Medicare Advantage, Medicare fee-for-service, Medicaid, other payer, or uninsured), visit type (ie, new or established), scheduled visit duration (10, 15, 20, or 30 minutes), diagnosis count (number of *ICD-10* diagnosis codes billed during the visit, a proxy for number of topics discussed), and chronic condition count. We used *ICD-10* codes and a 1-year look-back period to replicate widely used algorithms for 27 possible chronic condition categories.^28^

### Statistical Analysis

**Figure 1.**
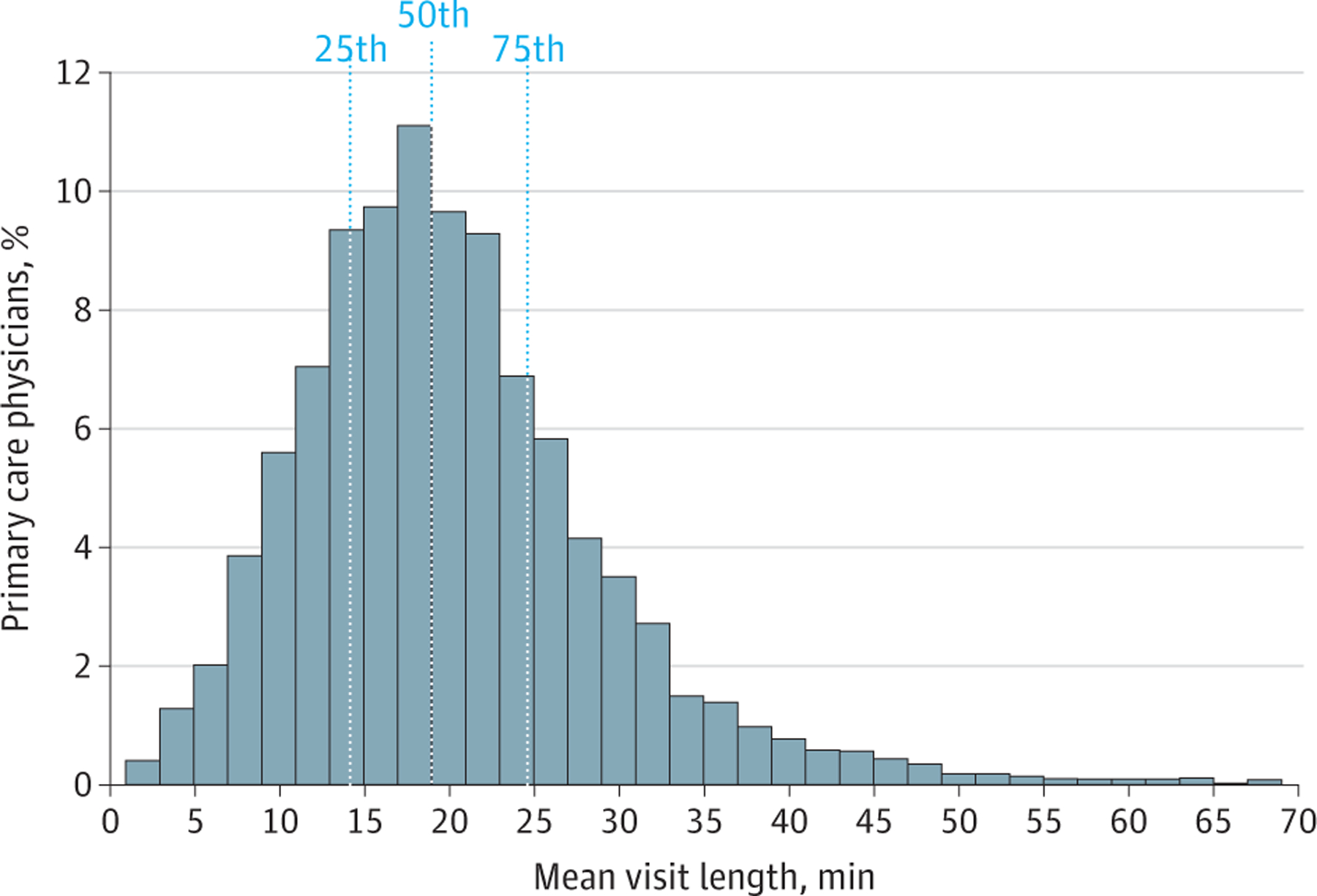
Histogram of Primary Care Physician Mean Visit Length During the 2017 Study Period Dotted lines denote the mean visit length for the 25th percentile, 50th percentile (median), and 75th percentile primary care physician.

Data were analyzed from March 2022 through January 2023. For each primary care physician included in the sample, we calculated their mean visit length and plotted a histogram (Figure 1) showing the proportion of physicians by the mean visit length. We used bivariate analyses to assess the association between patient and visit characteristics and visit length and then constructed a multivariable linear probability model with visit length as the outcome that included these characteristics. We then built a multivariable linear probability model to assess the association of visit length with potentially inappropriate prescribing, controlling for patient and visit characteristics. All models also included physician fixed effects to control for time-invariant differences across physicians in visit length and prescribing patterns. As such, results can be interpreted as comparisons of prescribing outcomes as a function of each individual physician’s variation in visit length. All inappropriate prescribing models were limited to visits with a length of 5 minutes or longer to exclude visits in which patient conditions may not have been discussed (eg, visits solely for prescription refills). All regression analyses used Huber-White robust SEs to assess statistical significance, which was defined as 2-sided *P* < .05. Analyses were conducted using Stata, version 16 (StataCorp LLC). To display adjusted regression results graphically, we used the binscatter command in Stata, which creates a binned scatterplot (ie, a nonparametric method of quantifying the mean y-value for equal-sized bins of x-values, controlling for patient and visit characteristics and including physician fixed effects).

## Results

The study sample consisted of 8 119 161 visits for 4 360 445 patients (43.4% men and 56.6% women) seeing 8091 primary care physicians in 4597 practices. Of the total visits, 7.7% were for Hispanic patients, 10.4% for non-Hispanic Black patients, 68.2% for non-Hispanic White patients, 5.5% for patients with other race and ethnicity, and 8.3% for patients with missing race and ethnicity (Table). Compared with a national sample of 8906 patients with office-based primary care visits from the National Ambulatory Medical Care Survey (NAMCS), patients receiving visits in the study sample were less likely to be non-Hispanic White (75.1% vs 68.2%), more likely to have commercial insurance (44.1% vs 48.5%) and Medicare (34.9% vs 40.2%), less likely to have Medicaid (8.3% vs 7.7%) or be uninsured (4.9% vs 2.6%), and more likely to have no chronic conditions (34.1% vs 41.5%) (eTable 2 in Supplement 1). Comparing the NAMCS sample with the athenahealth sample, we found similar rates of 1 chronic condition (24.5% vs 24.4%) and 2 chronic conditions (16.8% vs 16.4%), a similar sex distribution of visits, and a similar age distribution of visits (eTable 2 in Supplement 1). Compared with the NAMCS, the athenahealth sample somewhat overrepresented visits in the South (43.9% vs 54.9%) and underrepresented visits in the West (21.8% vs 8.6%), with similar proportions of visits in the Northeast (17.6% vs 17.7%) and Midwest (16.8% vs 18.7%). Patient and visit characteristics varied across the 3 subsamples used for our 3 potentially inappropriate prescribing measures (Table).

Visit duration varied considerably between and within primary care physicians. The median physician in the sample spent a mean of 18.9 minutes with each patient (Figure 1). Physicians in the top quartile of visit length spent a mean of 24.6 minutes or longer with their patients, while physicians in the bottom quartile of visit length spent a mean of 14.1 minutes or less with their patients.

### Factors Associated With Visit Length

**Figure 2.**
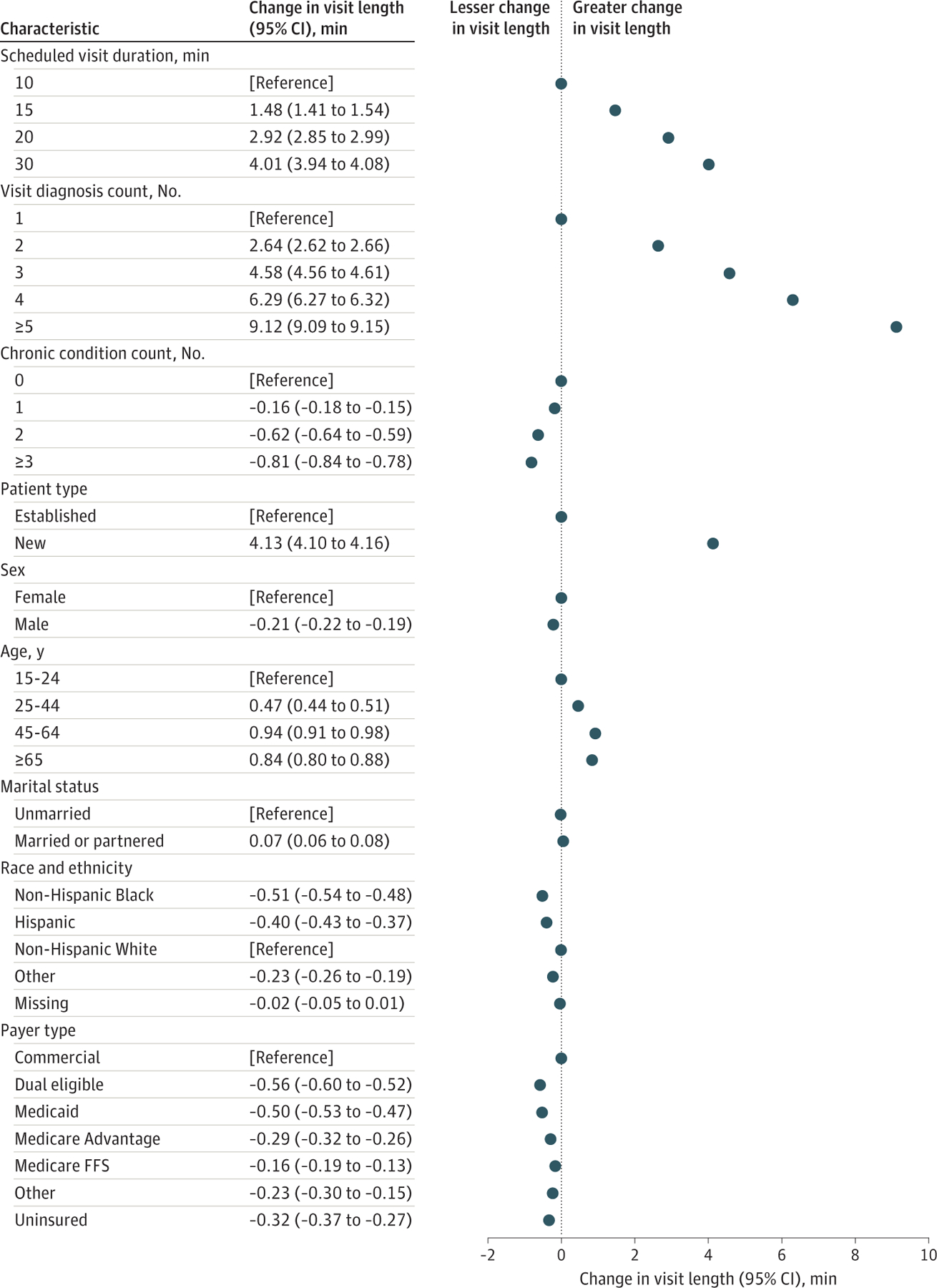
Factors Associated With Within-Physician Variation in Visit Length in 2017 Coefficients and 95% CIs were from a multivariable model including physician fixed effects and all patient or visit characteristics. Markers indicate the change in mean visit length associated with each patient or visit characteristic compared with the reference category, and whiskers indicate 95% CIs, which are too small to see due to the large sample size. eTable 3 in Supplement 1 shows a bivariate model regressing visit length by each characteristic individually. Other race and ethnicity includes American Indian or Alaska Native, Asian, and Native Hawaiian or Other Pacific Islander. Chronic condition count was based on the number of *International Statistical Classification of Diseases and Related Health Problems, Tenth Revision* (*ICD-10*) codes and a 1-year look-back period. Visit diagnosis count was calculated as the number of *ICD-10* diagnosis codes billed during the visit. FFS indicates fee for service.

When examining within-physician variation in visit length, we found that visit length was significantly associated with nearly every patient and visit characteristic (Figure 2 and eTable 3 in Supplement 1). Compared with a 10-minute scheduled visit, visits scheduled for 30 minutes received 4.0 additional minutes (95% CI, 3.9–4.1 minutes). Compared with visits with only 1 recorded diagnosis, visits with 5 or more diagnoses were 9.1 minutes (95% CI, 9.1–9.2 minutes) longer. Compared with visits for established patients, visits for new patients were 4.1 minutes (95% CI, 4.1–4.2 minutes) longer. Visit length was also slightly longer for female patients compared with male patients (female: 17.2 minutes [95% CI, 17.2–17.2 minutes]; male: 17.0 minutes [95% CI, 16.9–17.0 minutes]), patients aged 65 years or older compared with the youngest age groups (eg, ≥65 years: 17.2 minutes [95% CI, 17.1–17.2 minutes]; 25–44 years: 16.8 minutes [95% CI, 16.8–16.8 minutes]), non-Hispanic White patients compared with Hispanic and non-Hispanic Black patients and patients from other race and ethnicity (non-Hispanic White: 17.2 minutes [95% CI, 17.2–17.2 minutes]; Hispanic: 16.8 minutes [95% CI, 16.7–16.8 minutes]; non-Hispanic Black: 16.7 minutes [95% CI, 16.6–16.7 minutes]; and other: 16.9 minutes [95% CI, 16.9–17.0 minutes]), and patients with commercial insurance compared with all other types of insurance (eg, commercial: 17.2 minutes [95% CI, 17.2–17.2 minutes]; Medicaid: 16.7 minutes [95% CI, 16.7–16.8 minutes]).

### Prescribing Decisions

**Figure 3.**
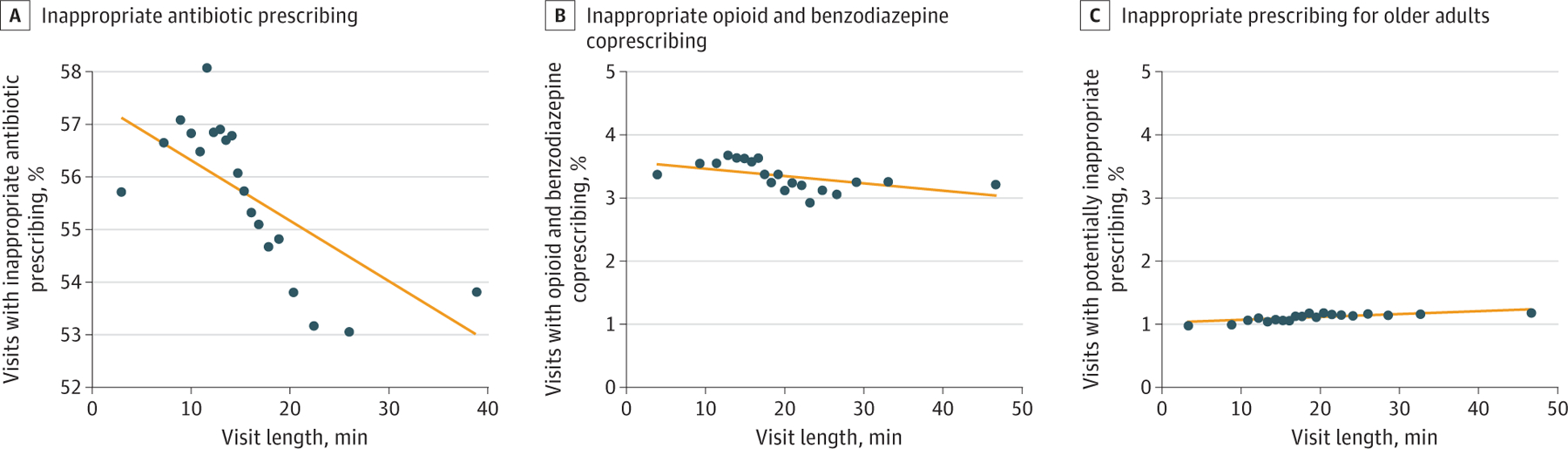
Association of Potentially Inappropriate Prescribing With Visit Length in 2017 Adjusted binned scatterplots and linear fit lines used ordinary least squares (OLS) regression. Dots indicate the mean y-value for equal-sized bins of x-values, controlling for patient and visit characteristics and including physician fixed effects. The following regression coefficients and 95% CIs were derived from the identical multivariable OLS model treating visit length as a continuous variable and including physician fixed effects and all patient or visit characteristics: A, −0.11 percentage points (95% CI, −0.14 to −0.09 percentage points); B, −0.01 percentage points (95% CI, −0.01 to −0.009 percentage points); and C, 0.004 percentage points (95% CI, 0.003–0.006 percentage points). C, Based on the Beers criteria.^27^

Within the study sample, 55.7% of 222 667 visits for upper respiratory tract infection involved an inappropriate antibiotic prescription, 3.4% of 1 571 935 visits for painful conditions involved coprescribing opioids and benzodiazepines, and 1.1% of 2 756 365 visits for adults aged 65 years or older involved the prescription of medications contraindicated by the Beers criteria. After adjusting for all patient covariates, the likelihood that an upper respiratory tract infection visit included an inappropriate antibiotic prescription decreased as visit length increased (Figure 3). For every additional minute of visit length, the likelihood of inappropriate antibiotic prescribing changed by −0.11 percentage points (95% CI, −0.14 to −0.09 percentage points) and the likelihood of opioid and benzodiazepine coprescribing changed by −0.01 percentage points (95% CI, −0.01 to −0.009 percentage points). In a sensitivity analysis limiting the sample of painful condition visits to those that also had an anxiety diagnosis, the likelihood of opioid and benzodiazepine coprescribing changed by −0.05 percentage points (95% CI, −0.07 to −0.04 percentage points) for every additional minute (eFigure 2 in Supplement 1). Potentially inappropriate prescribing among older adults increased slightly as a function of visit length (0.004 percentage points; 95% CI, 0.003–0.006 percentage points).

## Discussion

In a large, multistate sample of primary care visits, we found an association between visit length and some potentially inappropriate prescribing measures. When controlling for differences in physician practice style and patient and visit characteristics, longer visits were less likely to include an inappropriate prescription for an antibiotic and slightly less likely to include coprescribing of opioids and benzodiazepines. However, there was a positive association between visit length and prescribing a collection of potentially inappropriate medications for older adults that was unlikely to be clinically meaningful. This pattern of findings may reflect that inappropriate antibiotic prescribing would likely occur during acute care visits focusing on upper respiratory tract infection symptoms for which any additional time in the visit would likely be devoted to that single issue. In contrast, the other potentially inappropriate prescribing outcomes that we assessed are not specific to an acute condition (eg, coprescribing may occur for both acute and chronic pain) and therefore may occur in visits covering a range of patient concerns for which any additional time during the visit would not necessarily be allocated to the problem relevant to the potentially inappropriate prescribing outcome. For coprescribing and older adult outcomes, many of the prescriptions that we observed may have been refills; thus, it may have taken the physician less time to refill the medication than to engage in a discussion about deprescribing.

Given that shorter visit length was associated with some risk of lower-quality care, we were particularly interested in patient and visit characteristics that were associated with time spent with the physician. Many of these associations suggest that patients with more medical complexity or with more to discuss received more time with their physicians, which may be expected. For example, visits that included more diagnoses—an imperfect proxy for number of topics discussed—were longer, as were visits for patients with more previously recorded chronic conditions and for new patients. Interestingly, while visits with longer scheduled durations had longer observed durations, this was not a 1-to-1 association; visits scheduled for 30 minutes were only 4 minutes longer than those scheduled for 10 minutes, which tended to run longer than their scheduled time. This finding suggests that scheduled visit times do not necessarily represent clinical workflows accurately and points to the challenges that primary care physicians may face in adhering to scheduled visit times to care for a wide range of patients with diverse needs.

We also have particular concerns about the associations we found between patient-visit characteristics and visit length that were not easily explained by differences in perceived patient clinical need. For example, patients with Medicaid insurance coverage, dual Medicare and Medicaid coverage, or no insurance coverage received significantly shorter visits than commercially insured patients despite the latter population being healthier on average. Similarly, non-Hispanic Black patients received visits that were shorter, on average, than non-Hispanic White patients seeing the same physician. These visit-level differences may accumulate over time, potentially contributing to racial disparities in how much time patients spend with their physicians each year.^29^ Our analyses cannot explain why these differences exist but should motivate organizations and policy makers to detect, interrogate, and address underlying systemic causes such as structural racism.^30^

Our analyses highlight the fundamental tension between the volume incentives embedded in fee-for-service reimbursement systems and quality of care.^25,31–33^ While our results do not suggest an optimal visit length, they do suggest that physicians’ time is not always allocated based on patient complexity.^34^ Such findings suggest opportunities for a more equitable distribution. While risk adjusting visit length to match individual patients’ needs may be prohibitively complex from a logistical standpoint, practice leads could consider building in more flexibility than typically exists now. For example, practices could allow for 2 different visit lengths for problem-based visits, enabling physicians to indicate in advance which patients would benefit from the longer visit.

In particular, policy makers and health system leaders wishing to advance antibiotic stewardship best practices should take note of the association between visit length and inappropriate antibiotic prescribing. Our findings suggest that lengthening upper respiratory tract infection visits may be a promising strategy to lower inappropriate antibiotic prescribing, which has been a persistent population health concern for decades. However, meaningful gains in improved patient care quality and safety require that increases in visit time be accompanied by other thoughtful implementation strategies (eg, decision supports and shared decision-making tools) that promote consistency in value-based decision-making.

### Limitations

This study has several limitations. First, the results of this study should not be interpreted causally, although we were able to improve on existing studies of associations by comparing within-physician (rather than across-physician) variation in visit length and associated prescribing outcomes. There could still be unobserved reasons (eg, different communication styles, language barriers) for why patients were less likely to receive inappropriate antibiotic prescriptions during longer visits. Second, the cross-sectional nature of our data mean that we were unable to examine changes over time in prescribing patterns. Third, we relied on data from a convenience sample of primary care physicians who chose to purchase the services of athenahealth, and therefore our results may not generalize to all primary care physicians in the US. However, in many respects, primary care physicians within the athenahealth network appear to resemble primary care physicians in the US. Fourth, the samples that relied on diagnosis codes (ie, inappropriate antibiotic prescribing) likely changed with visit length since physicians may code themselves out of inappropriate antibiotic prescribing by recording different diagnoses during longer visits. Relatedly, visit length and diagnosis codes are an imperfect proxy for what was discussed by patients and physicians during the encounter. Fifth, our measure of opioid-benzodiazepine coprescribing was likely an underestimate since we defined it as coprescribing within the same visit. Patients may have an active opioid prescription when they receive a benzodiazepine prescription and vice versa. Sixth and relatedly, the potentially inappropriate prescribing decisions that we captured represent a small subset of the overall quality of care provided by a physician. Notably, we were unable to examine physician decisions indicative of high-quality (rather than low-quality) care, nor did we examine other facets of primary care quality such as referral decisions or diagnostic accuracy, which may also be associated with visit length.

## Conclusions

In this cross-sectional study of primary care physician visit length, shorter visit length was associated with higher rates of inappropriate antibiotic prescribing for upper respiratory tract infections and inappropriate coprescribing of opioids and benzodiazepines for patients with painful conditions, but similar patterns were not found for other potentially inappropriate prescribing decisions. We found considerable within-physician variation in visit length, with younger, publicly insured, Hispanic, and non-Hispanic Black patients receiving shorter visits. These findings suggest opportunities for additional research and operational improvements to visit scheduling and quality of prescribing decisions in primary care.

## Supplementary Material

Supp info SUPPLEMENT 1.
**eFigure 1.** Sample Selection Diagram
**eTable 1.** *ICD-10* Diagnoses Used to Define Subsamples Relevant to Potentially Inappropriate Prescribing Outcomes
**eTable 2.** Patient and Appointment Characteristics, Within the athenahealth Sample and the National Ambulatory Medical Care Survey (NAMCS)
**eTable 3.** Bivariate and Multivariate Exam Length Regression Results
**eFigure 2.** Association of Opioid and Benzodiazepine Coprescribing With Visit Length, in Visits With a Painful Condition and Anxiety Diagnosis, 2017

Data Sharing Statement SUPPLEMENT 2.
Data Sharing Statement

## Figures and Tables

**Table.** Patient and Visit Characteristics for athenahealth Inc Primary Care Visit Samples in 2017^a^

Characteristic	Visits, No. (%)^b^			
	All visits (N = 8 119 161)	URTI visits (n = 222 667)	Painful condition visits (n = 1 571 935)	Visits for adults aged ≥65 y (n = 2 756 365)
Patient characteristics				
Age, y				
15–24	423 159 (5.2)	13 791 (6.2)	38 780 (2.5)	NA
25–44	1 549 904 (19.1)	46 795 (21.0)	263 784 (16.8)	NA
45–64	3 018 012 (37.2)	85 158 (38.2)	665 600 (42.3)	NA
≥65	3 128 086 (38.5)	76 923 (34.6)	603 771 (38.4)	2 756 365 (100)
Se x				
Female	4 659 341 (57.4)	137 497 (61.8)	955 837 (60.8)	1 601 126 (58.1)
Male	3 459 820 (42.6)	85 170 (38.2)	616 098 (39.2)	1 155 234 (41.9)
Race and ethnicity				
Hispanic	625 388 (7.7)	17 577 (7.9)	121 250 (7.7)	141 840 (5.2)
Non-Hispanic Black	842 085 (10.4)	20 570 (9.2)	184 714 (11.8)	223 750 (8.1)
Non-Hispanic White	5 533 973 (68.2)	152 383 (68.4)	1 070 344 (68.1)	2 091 428 (75.9)
Other^c^	443 908 (5.5)	13 281 (6.0)	76 386 (4.9)	116 831 (4.2)
Missing	673 807 (8.3)	18 856 (8.5)	119 241 (7.6)	182, 516 (6.6)
Marital status				
Unmarried	3 789 506 (46.7)	99 877 (44.8)	747 638 (47.6)	1 187 144 (43.1)
Married or partnered	4 329 655 (53.3)	122 790 (55.2)	824 297 (52.4)	1 569 221 (56.9)
Insurance primary payer				
Commercial	3 941 416 (48.5)	121 357 (54.5)	675 907 (43.0)	255 208 (9.3)
Dual eligible	388 330 (4.8)	8740 (3.9)	106 726 (6.8)	174 666 (6.3)
Medicaid	628 305 (7.7)	15 796 (7.1)	155 285 (9.9)	14 679 (0.5)
Medicare Advantage	946 068 (11.7)	22 443 (10.1)	198 295 (12.6)	724 228 (26.3)
Medicare FFS	1 928 771 (23.8)	47 700 (21.4)	376 309 (23.9)	1 571 469 (57.0)
Other	79 405 (1.0)	1157 (0.5)	22 351 (1.4)	7860 (0.3)
Uninsured	206 866 (2.6)	5474 (2.5)	37 062 (2.4)	8255 (0.3)
Chronic condition count^d^				
0	3 366 861 (41.5)	146 957 (66.0)	582 501 (37.1)	645 603 (23.4)
1	1 976 796 (24.4)	41 016 (18.4)	397 857 (25.3)	633 365 (23.0)
2	1 335 018 (16.4)	19 454 (8.7)	280 744 (17.1)	612 243 (22.2)
≥3	1 440 486 (17.7)	15 240 (6.8)	310 833 (19.8)	865 154 (31.4)
**Visit characteristics**				
Scheduled duration, min				
10	664 327 (8.2)	20 723 (9.3)	126 366 (8.0)	195 383 (7.1)
15	5 569 710 (68.6)	157 626 (70.8)	1 067 364 (67.9)	1 887 890 (68.5)
20	1 011 353 (12.5)	25 765 (11.6)	210 331 (13.4)	348 795 (12.7)
30	873 771 (10.8)	18 553 (8.3)	167 874 (10.7)	324 297 (11.8)
Visit type				
New patient	436 489 (5.4)	11 397 (5.1)	89 810 (5.7)	87 100 (3.2)
Established patient	7 682 672 (94.6)	211 270 (94.9)	1 482 125 (94.3)	2 669 265 (96.8)
Visit diagnosis count^e^				
1	1 429 915 (17.6)	64 962 (29.2)	135 274 (8.6)	306 873 (11.1)
2	1 382 945 (17.0)	54 092 (24.3)	196 881 (12.5)	348 952 (12.7)
3	1 249 823 (15.4)	34 930 (15.7)	220 278 (14.0)	375 281 (13.6)
4	1 379 106 (17.0)	27 593 (12.4)	281 949 (17.9)	510 961 (18.5)
≥5	2 677 372 (33.0)	41 090 (18.5)	737 553 (46.9)	1 214 298 (44.1)

Abbreviations: FFS, fee-for-service; *ICD-10, International Statistical Classification of Diseases and Related Health Problems, Tenth Revision*; NA, not applicable; URTI, upper respiratory tract infection.

a There were 4 360 445 patients for all visits, 206 307 patients with URTI visits, 1 074 437 patients with painful condition visits, and 1 379 393 adults aged 65 years or older with visits.

b eTable 1 in Supplement 1 gives sample-defining diagnosis codes; samples of URTI visits, painful visits, and visits for patients aged 65 years or older were limited to visits 5 minutes or longer.

c Other includes American Indian or Alaska Native, Asian, and Native Hawaiian or Other Pacific Islander.

d Number based on *ICD-10* codes and a 1-year look-back period.

e Number of *ICD-10* diagnosis codes billed during the visit.

## Key Points

### Question

Are primary care physicians more likely to prescribe potentially inappropriate medications during shorter visits?

### Findings

In this cross-sectional study of 4 360 445 patients, those who were younger, publicly insured, Hispanic, or non-Hispanic Black had shorter primary care physician visits. Shorter visits were associated with a higher likelihood of inappropriate antibiotic prescribing for patients with upper respiratory tract infections and coprescribing of opioids and benzodiazepines for patients with painful conditions.

### Meaning

In this study, shorter primary care visits were associated with some, but not all, measures of inappropriate prescribing.
