# Supplementary material for: Association of Primary Care Visit Length With Potentially Inappropriate Prescribing
Source: JAMA Health Forum. Author manuscript; Available in PMC 2024 Mar 3. (PMC10249052; doi:10.1001/jamahealthforum.2023.0052)
Supplement: Supp info — SUPPLEMENT 1. eFigure 1. Sample Selection Diagram eTable 1. ICD-10 Diagnoses Used to Define Subsamples Relevant to Potentially Inappropriate Prescribing Outcomes eTable 2. Patient and Appointment Characteristics, Within the athenahealth Sample and the National Ambulatory Medical Care Survey (NAMCS) eTable 3. Bivariate and Multivariate Exam Length Regression Results eFigure 2. Association of Opioid and Benzodiazepine Coprescribing With Visit Length, in Visits With a Painful Condition and Anxiety Diagnosis, 2017 [file NIHMS1894389-supplement-Supp_info.pdf]

Supplemental Online Content

Neprash HT, Mulcahy JF, Cross DA, Gaugler JE, Golberstein E, Ganguli I. Association of primary care visit length with potentially inappropriate prescribing. *JAMA Health Forum*. 2023;4(3):e230052. doi:10.1001/jamahealthforum.2023.0052

eFigure 1. Sample Selection Diagram

eTable 1. *ICD-10* Diagnoses Used to Define Subsamples Relevant to Potentially Inappropriate Prescribing Outcomes

eTable 2. Patient and Appointment Characteristics, Within the athenahealth Sample and the National Ambulatory Medical Care Survey (NAMCS)

eTable 3. Bivariate and Multivariate Exam Length Regression Results

eFigure 2. Association of Opioid and Benzodiazepine Coprescribing With Visit Length, in Visits With a Painful Condition and Anxiety Diagnosis, 2017

This supplementary material has been provided by the authors to give readers additional information about their work.

© 2023 Neprash HT et al. *JAMA Health Forum*.

eFigure 1. Sample Selection Diagram

Main visit sample (N=14,815,881)
Office or hospital outpatient department visits in 2017 to primary care physicians (internal medicine, family practice, general practice) who used the athenahealth electronic health record and its eprescribing functionality, for commonly schedule visit durations (10-, 15-, 20-, and 30-minutes) and patients aged ≥15.

Drop visits with start and stop timestamps on different days **(N=14,395,022)**

Drop visits with a start timestamp >2 hours after scheduled start time **(N=14,025,155)**

Drop visits with a stop timestamp >2 hours after scheduled stop time **(N=13,608,854)**

Drop visits where exam timestamps overlap with another exam start and stop timestamps **(N=8,119,161)**

© 2023 Neprash HT et al. *JAMA Health Forum*.

eTable 1. *ICD-10* Diagnoses Used to Define Subsamples Relevant to Potentially Inappropriate Prescribing Outcomes

Potentially Inappropriate Prescribing Measure	Primary Care Visit Sample	Specifications
Inappropriate antibiotic prescribing	Visits with upper respiratory infection as the primary diagnosis	ICD-10 Diagnoses: J00, J04.0, J04.2, J05.0, J06, J06.0, J06.9, J09.X, J09.X3, J10.2, J10.8, J10.81, J10.82, J10.89, J11.2, J11.8, J11.81, J11.82, J11.89, J12, J12.0, J12.1, J12.2, J12.3, J12.8, J12.81, J12.89, J12.9, J20, J20.0, J20.1, J20.2, J20.3, J20.4, J20.5, J20.6, J20.7, J20.8, J20.9, J21, J21.0, J21.1, J21.8, J21.9
Co-prescribing of opioids and benzodiazepines	Visits for back pain, joint pain, musculoskeletal pain, migraine, and other pain	ICD-10 Diagnoses: Back pain (F45.42, M25.78, M43.2x, M43.6, M43.8X9, M46.4x, M47.x, M48.0x, M48.1x, M48.2x, M48.3x, M48.9, M50.x, M51.x, M53.0, M53.1, M53.2X7, M53.2X8, M53.3, M53.8x, M53.9, M54.0x, M54.11-M54.17, M54.2, M54.3x, M54.4x, M54.5, M54.6, M54.8x, M54.9, M62.830, M96.1, M99.2x, M99.3x, M99.4x, M99.5x, M99.6x, M99.7x, S134XXA, S138XXA, S139XXA, S161XXA, S233XXA, S238XXA, S239XXA, S335XXA, S336XXA, S338XXA, S339XXA), joint pain (A18.01, A18.02, A52.16, E08.610, E08.618, E09.610, E09.618, E10.610, E10.618, E11.610, E11.618, E13.610, E13.618, M00.x, M01.Xx, M02.x, M05.x, M06.x, M07.6x, M08.x, M11.x, M12.x, M13.x, M14.x, M15.x, M16.x, M17.x, M18.x, M19.x, M22.x, M23.x, M24.0x, M24.1x, M24.3x, M24.4x, M24.5x, M24.6x, M24.7, M24.8x, M24.9, M25.0x, M25.1x, M25.2x, M25.3x, M25.4x, M25.5x, M25.6x, M25.8x, M25.9, M32.x, M33.x, M34.x, M35.0x, M35.1, M35.2, M35.5, M35.8, M35.9, M36.x, M43.3, M43.4, M43.5Xx, M45.x, M46.0x, M46.1, M46.5x, M46.8x, M46.9x, M48.8x, M49.8x, M53.2X1-M53.2X6, M53.2X9, M79.646, Q68.6, R26.2, R29.4), musculoskeletal pain (M20.10, M24.2x, M25.70, M25.71x, M25.72x, M25.73x, M25.74x, M25.75x, M25.76x, M25.77x, M35.4, M35.6, M35.7, M54.10, M54.18, M60.x, M61.x, M62.0x, M62.1x, M62.2x, M62.3, M62.4x, M62.5x, M62.81, M62.82, M62.831, M62.838, M62.84, M62.89, M62.9, M63.x, M65.x, M66.x, M67.x, M70.x, M71.x, M72.x, M75.x, M76.x, M77.x, M79.x, R25.2, R29.898), migraine (G43.x), other pain (G44.209, G89.21, G89.22, G89.28, G89.29, G89.4, M35.3)

© 2023 Neprash HT et al. *JAMA Health Forum*.

Potentially inappropriate prescribing among older adults (Beers Criteria)	Visits for adults aged 65+	Qualifying medications:<br>Hydroxyzine and promethazine;<br>Nifedipine;<br>Tertiary Tricyclic Antidepressants [Amitriptyline, Chlordiazepoxide-amitriptyline, Clomipramine, Doxepin > 6 mg/d, Imipramine, Perphenazine-amitriptyline, Trimipramine];<br>Barbiturates [Amobarbital, Butabarbital, Butalbital, Mephobarbital, Pentobarbital, Phenobarbital, Secobarbital];<br>Ergot mesylates [Isoxsuprine];<br>Long duration Sulfonylureas [Chlorpropamide, Glyburide];<br>Meperidine

© 2023 Neprash HT et al. *JAMA Health Forum*.

eTable 2. Patient and Appointment Characteristics, Within the athenahealth Sample and the National Ambulatory Medical Care Survey (NAMCS)

	NAMCS (2018)	athenahealth sample (2017)
Chronic Condition Count		
0	34.1%	41.5%
1	24.5	24.4
2	16.8	16.4
3+	22.1	17.7
Missing	2.5	0
Sex		
Female	57.8	57.4
Male	42.3	42.6
Age Category		
15-24	7.3	5.2
25-44	19.1	19.1
45-64	31.6	37.2
65+	42	38.5
Race/ethnicity		
Black, Non-Hispanic	6.7	10.4
Hispanic	12.5	7.7
White, Non-Hispanic	75.1	68.2
Other	5.7	5.5
Missing	NA	8.3
Insurance Primary Payer		
Commercial	44.1	48.5
Medicare	34.9	40.2
Medicaid	8.3	7.7
Uninsured	4.9	2.6
Other Payer	1.8	1
Missing	5.9	0
Geographic Region		
Midwest	16.8	18.7
Northeast	17.6	17.7
South	43.9	54.9
West	21.8	8.6

SOURCE: Authors' analysis of athenahealth and NAMCS data

© 2023 Neprash HT et al. *JAMA Health Forum*.

NOTE: Percentages may not sum to 100, due to rounding. Both samples include all recorded primary care physician office visits. NAMCS uses imputation to create complete race ethnicity data. 18.9% of cases have both race and ethnicity imputed, 8.1% of cases have only race imputed, and 7.4% of cases have only ethnicity imputed. Geographic region comes from the 2016 NAMCS Summary Web Tables (Table 13), since more recent NAMCS public use files do not contain this information.

© 2023 Neprash HT et al. *JAMA Health Forum*.

eTable 3. Bivariate and Multivariate Exam Length Regression Results

	Bivariate Models		Multivariate Model	
	Coefficient	95% CI	Coefficient	95% CI
Scheduled Duration (Ref = 10 minutes)				
15 minutes	2.28	2.21, 2.35	1.48	1.41, 1.54
20 minutes	4.36	4.28, 4.43	2.92	2.85, 2.99
30 minutes	5.97	5.90, 6.04	4.01	3.94, 4.08
Visit Diagnosis Count (Ref = 1)				
2	2.65	2.63, 2.68	2.64	2.62, 2.66
3	4.54	4.52, 4.57	4.58	4.56, 4.61
4	6.17	6.14, 6.19	6.29	6.27, 6.32
5+	8.92	8.89, 8.94	9.12	9.09, 9.15
Chronic Conditions (Ref = 0)				
1	2.00	1.98, 2.02	-0.16	-.18, -.15
2	3.11	3.09, 3.13	-0.62	-.64, -.59
3+	3.92	3.89, 3.94	-0.81	-.84, -.78
New Patient (Ref = Established Patient)	4.56	4.52, 4.59	4.13	4.10, 4.16
Male Gender (Ref = Female)	-0.11	-.12, -.09	-0.21	-.22, -.19
Age Group (Ref = 15 - 24)				
25 - 44	1.21	1.18, 1.25	0.47	.44, .51
45 - 64	2.44	2.41, 2.47	0.94	.91, .98
65+	2.50	2.47, 2.54	0.84	.80, .88
Married/ Partnered (Ref = Not Married)	0.25	.24, .27	0.07	.06, .08
Race Category (Ref = white, non-Hispanic)				
Black, Non-Hispanic	-0.46	-.49, -.44	-0.51	-.54, -.48
Hispanic	-0.58	-.61, -.54	-0.40	-.43, -.37
Other	-0.32	-.35, -.29	-0.23	-.26, -.19
Missing	-0.005	-.04, .03	-0.02	-.05, .01
Payer Type (Ref = Commercial)				
Dual Eligible	0.19	.16, .23	-0.56	-.60, -.52
Medicaid	-0.42	-.45, -.39	-0.50	-.53, -.47
Medicare Advantage	0.53	.51, .55	-0.29	-.32, -.26
Medicare FFS	0.53	.51, .55	-0.16	-.19, -.13
Other Payer	-0.87	-.95, -.79	-0.23	-.30, -.15
Uninsured	-0.69	-.74, -.64	-0.32	-.37, -.27

SOURCE: Authors' analysis of athenahealth data

NOTE: Bivariate and multivariate models include physician fixed effects.

© 2023 Neprash HT et al. *JAMA Health Forum*.

eFigure 2. Association of Opioid and Benzodiazepine Coprescribing With Visit Length, in Visits With a Painful Condition and Anxiety Diagnosis, 2017

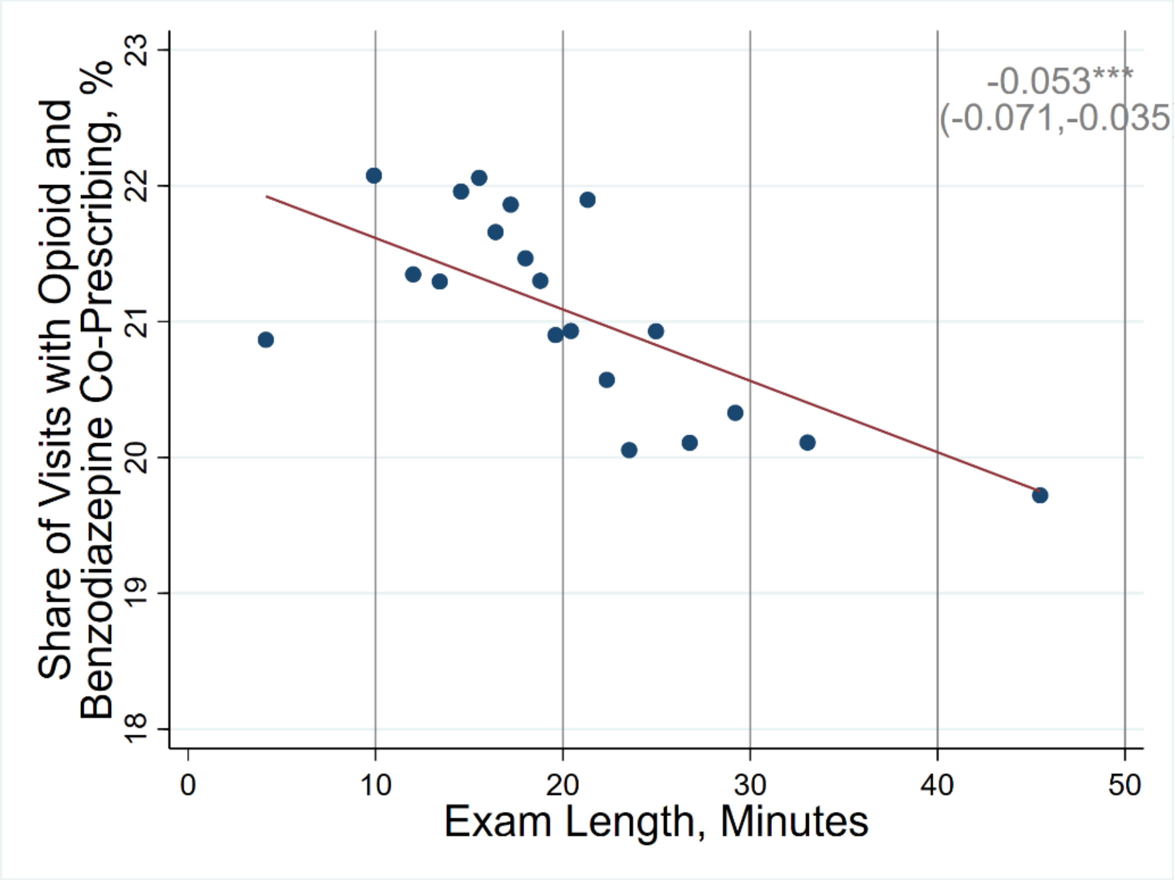

Note: This figure displays an adjusted binned scatterplot and linear fit line using ordinary least squares (OLS) regression. Each dot displays the average y-value for equal-sized bins of x-values, controlling for patient and visit characteristics described in the text and including physician fixed effects. The regression coefficient and 95% confidence interval in the top right derives from the identical multivariate OLS model treating visit length as a continuous variable and including physician fixed effects and all patient/visit characteristics.

© 2023 Neprash HT et al. *JAMA Health Forum*.
