# Supplementary material for: Association of Primary Care Visit Length With Potentially Inappropriate Prescribing
Source: JAMA Health Forum. Author manuscript; Available in PMC 2024 Mar 3. (PMC10249052; doi:10.1001/jamahealthforum.2023.0052)
Supplement: Data Sharing Statement — SUPPLEMENT 2. Data Sharing Statement [file NIHMS1894389-supplement-Data_Sharing_Statement.pdf]

Data Sharing Statement

Neprash. Association of Primary Care Visit Length With Potentially Inappropriate Prescribing. *JAMA Health Forum*. Published March 10, 2023. doi:10.1001/jamahealthforum.2023.0052

Data
Data available: No

Additional Information
Explanation for why data not available: Our data use agreement does not permit data-sharing.
